# Kindness to Children

**Published:** 1881-12

**Authors:** 


Children possess a keenness of judgment and a delicacy of impression incredible to those who have not studied their characters. Justice and equity are easily born in their minds, for they are, above all things, absolutely logical. Take advantage of all this. There are unjust and harsh words that engrave themselves at the bottom of a child's heart and remain there his whole life long. Remember there is in your baby that which will some day be a man, whose love will be the light of your old age; respect him that he may respect you, and rest assured that not one seed, thrown into this little heart, will fail to produce its fruit.

---

It is well enough to travel in search of your health, but pray do not forget to bring it back with you on your return.

A recipe for lemon pie adds, vaguely: "Then sit on a stove and stir constantly." Just as if anybody could sit on a stove without stirring constantly.

### SANITARY DEPARTMENT OF "HALL'S JOURNAL OF HEALTH."

We are daily receiving requests from our readers to purchase for them "HEALTH FOODS," "FOOD MEDICINES," PURE BAKING POWDERS, and various MEDICINES that our experience has taught us the value of; also standard books; and last but not least, many families rely on us to procure for them PURE BOVINE VACCINE MATTER, which is the only vaccine matter that is always safe. These commissions already keep one person employed much of the time. We have therefore determined to make this a branch of our regular business, and announce ourselves ready to fill orders for GLUTEN OF WHEAT, GLUTEN COFFEE, COOKED FOODS, as CRUSHED WHEAT, BARLEY, OATS, CORN and RYE, also the various preparations of MALT, and for books, medicines, vaccine matter and any other like articles that can be found in New York market.

E. H. GIBBS & CO.

